# THE RELATIONSHIP BETWEEN FRACTURES IN PEDIATRIC POLYTRAUMA PATIENTS: EVALUATION OF CLINICAL OUTCOMES

**DOI:** 10.1590/1413-785220233103e268013

**Published:** 2023-07-17

**Authors:** DAVI BRAGA DE CARVALHO, EIFFEL TSUYOSHI DOBASHI, DANILO JOSÉ LEITE GOMES, JOSÉ MANOEL DANTAS, ANA JULIA MARQUEZ PAJUABA, LUIZ FERNANDO COCCO

**Affiliations:** 1 Universidade Federal de São Paulo, Escola Paulista de Medicina, São Paulo, SP, Brazil.

**Keywords:** Child, Child, Hospitalized, Child Mortality, Femoral Fractures, Pelvis, Pelvic Bones, Criança, Criança Hospitalizada, Mortalidade da Criança, Fraturas do Fêmur, Pelve, Ossos Pélvicos

## Abstract

**Objective:**

To evaluate children and adolescents with polytrauma and fractures of the pelvis and proximal and diaphyseal femur and correlate the impact of these conditions and clinical outcomes.

**Methods:**

Retrospective study carried out in a public hospital in Taboão da Serra (SP), with pediatric patients with polytrauma from January 2012 to December 2021. In total, 44 patients were evaluated, 70.44% boys and 29.55% girls, aged from 12 to 17 years.

**Results:**

Diaphyseal fracture of the femur affected 70.44% of the patients, mainly caused by a fall from a height (56.81%). Linear external fixation was the most used treatment (45.45%). All patients were discharged from hospital.

**Conclusion:**

We found essential sociodemographic information: 84.11% of patients did not have associated injuries; 88.63% were hospitalized from 3 to 11 days; 90.91% did not need to be admitted to an ICU, 77.27% did not need reoperation, and 22.73% underwent another surgery; 45.45% used the external fixator to stabilize injuries; 11.36% converted the external fixator to the intramedullary nail; 9.09% needed an intramedullary nail remover; 2.27% converted to a plate (bilateral) and 2.27% to a rigid nail; 2.27% had loss of reduction and revision with rod; 2.27% underwent corrective osteotomy; 2.27% had clinical hospitalization; 2.27% had osteonecrosis of the femoral head and screws removed; 2.27% removed the plate. No deaths were recorded. ***Level of Evidence II, Retrospective Study.***

## RESUMO

**Objetivo:**

Avaliar crianças e adolescentes politraumatizados com fraturas da pelve, proximal e diafisária do fêmur e correlacionar o impacto dessas condições e desfechos clínicos.

**Métodos:**

Estudo retrospectivo realizado em hospital público de Taboão da Serra (SP), com pacientes pediátricos politraumatizados entre janeiro de 2012 e dezembro de 2021. Avaliaram-se 44 pacientes, 70,44% meninos e 29,55% meninas, de 12 a 17 anos.

**Resultados:**

A fratura diafisária fechada do fêmur acometeu 70,44%, sendo causada principalmente por queda de altura (56,81%). A fixação externa linear foi o tratamento mais utilizado (45,45%). Todos os pacientes receberam alta hospitalar.

**Conclusão:**

Identificaram-se importantes informações sociodemográficas: 84,11% dos pacientes não apresentaram lesões associadas; 88,63% ficaram internados de 3 a 11 dias; 90,91% não necessitaram de internação em unidade de terapia intensiva (UTI), 77,27% não precisaram ser reoperados; 22,73% realizaram nova cirurgia; 45,45% utilizaram fixador externo para estabilização de lesões;11,36% converteram o fixador externo para a haste intramedular; 9,09% precisaram remover as hastes intramedulares; 2,27% converteram para placa (bilateral) e 2,27% para haste rígida; 2,27% tiveram perda de redução e revisão com haste; 2,27% realizaram osteotomia corretiva; 2,27% tiveram internação clínica; 2,27% tiveram osteonecrose da cabeça femoral e parafusos removidos; e 2,27% retiraram a placa. Não foram observados óbitos. **Nível de Evidência II, Estudo Retrospectivo.**

## INTRODUCTION

Trauma is characterized by structural changes or physiological imbalances resulting from acute exposure to various forms of kinetic energy.^1^ In immature skeleton, trauma injury is the leading cause of death worldwide, significantly hindering children’s development. ^(^ ^2^

According to the Committee on Trauma of the American College of Surgeons, at least 25% of all patients involved with trauma are children. ^(^ ^3^ The countries of Southeast Asia and the Western Pacific have the highest mortality rates from pediatric trauma worldwide. ^(^ ^4^ In Brazil, studies that stand out describing the epidemiology, prevalence, and potential risk factors in childhood and adolescent polytraumatism are scarce. ^(^ ^1^

Regarding the Brazilian population, the characteristics particular to children, as its proportions, shapes, size, volume, volume of the organs, among other peculiar ones, may increase or decrease the risk of trauma injuries. ^(^ ^5^ Other characteristics inherent to the child should also be valued such as sex, age group, region of trauma and manner in which they occurred, especially regarding neglect and child abuse. ^(^ ^2^

Notably, the severity of the injury, reflected by the Injury Severity Score (ISS), is estimated based on the Abbreviated Injury Scale (AIS) and considers the three regions of the body most severely injured. Regarding the ISS, ‘multiple injury occurs when its coefficient results in a value ≥ 16. ^(^ ^6^ ^),(^ ^7^

The incidence of fractures of the pelvic bones in the immature skeleton varies from 2.4 to 7.5% in the reference services for trauma. ^(^ ^4^ Although this lesion presents a low frequency, they result from accidents with high kinetic energy and represent 5% of all admissions to hospital facilities. They are also highly associated with multisystem impairments determined by a high severity score (ISS). ^(^ ^8^ ^),(^ ^9^

The efficient and early evaluation and management of these disorders are essential to improve the evolution of patients, determining the best clinical outcomes. This would reduce the length of hospital stay, sequelae, mortality rate, inherent socioeconomic problems, and treatment costs.

Physical examination, as well as imaging techniques-such as plain radiography, ultrasonography, and computed tomography-are crucial for the early diagnoses of the individual with trauma. Moreover, laboratory analyses are essential adjunctive tools for monitoring pediatric lesions. The alteration of inflammatory markers correlates directly with post-traumatic complications, including the recognition of multiple organ failure of the human body. ^(^ ^1^

The seriously injured child requires interdisciplinary intervention. Although many sources of clinical information, laboratory and imaging markers can be found, the lack of prospective controlled studies concomitantly using these resources prevents the creation of safe and applicable guidelines for pediatric trauma.

Several laboratory markers have recently been described to support the early diagnosis and prognosis of severely injured children. The concomitant application of imaging techniques is essential and combining these techniques with laboratory biomarkers has reliable prognostic value and can improve the evaluation of pediatric lesions after trauma. Specific inflammatory markers may be relevant for multiple organ evaluation, especially in post-traumatic complications. Lactate concentration, blood coagulation parameters, and specific markers of organ damage are crucial clinical tools in the early identification of trauma-induced disorders.

Severe trauma is the most common cause of mortality in children whom the most frequently injured structures are the head and the chest, followed by the extremities and abdomen. The former regions are associated with a high socioeconomic burden. The efficient and early evaluation and management of these lesions are essential for better evolution of patients.

Due to the continuous efforts and studies conducted on trauma care, the rate of infant mortality and disability significantly decreased. Regarding etiology, accidents involving high kinetic energy such as road traffic injuries are the main cause of mortality and disability worldwide.

This study aimed to evaluate the clinical outcome regarding the risk of death in patients with pediatric polytrauma with fractures of the pelvis and/or femur. Furthermore, this study evaluated the length of hospital stay, need for ICU admission, and treatment methods used.

## METHODS

The project of this study was initially submitted to the Research Ethics Committee of the Universidade Federal de São Paulo (UNIFESP) by the Plataforma Brasil, for the evaluation and request for exemption from the informed consent form, and was approved for implementation under CAAE number 5,612,632.

A quantitative, cross-sectional, retrospective, and documentary study was performed by descriptively analyzing the data collected. The study was conducted at the Hospital of Pirajussara, inaugurated in 1999. The hospital is a reference for about 570,187 people who live in the region, which comprises the municipalities of Embu das Artes and Taboão da Serra, in the state of São Paulo. Moreover, the hospital interacts with the Municipal Health Systems in line with the principles of universality, regionalization, and hierarchization of the Brazilian Unified Health System (SUS). All traumatic injuries of children and adolescents from these regions that require orthopedic evaluation with hospitalization and treatment are referred to the hospital.

The target population of this study consisted of all pediatric patients diagnosed with polytrauma who also had proximal femur fractures, diaphyseal femur fractures, and/or pelvic ring fractures. The study period was from January 2012 to December 2021.

After the analysis of the entire database of the hospital, a sample of 44 patients was obtained. In total, 31 (70.45%) were male and 13 (29.55%) female. When analyzing participants’ ages, 11 (25.00%) individuals were aged from 1 to 7 years, 5 (11.36%) aged from 8 to 11 years, and 28 (63.64%) aged from 12 to 17 years (Tables 1 and 2).

**Table 1:** Distribution of patients according to sex.

Sex	Patients	Percentage (%)
Male	31	70.45
Female	13	29.55
Total	44	100.00

**Table 2:** Distribution of patients according to age groups

Age	Patients	Percentage (%)
0-7	11	25.00
8-11	5	11.36
12-17	28	63.64

To identify the municipalities of origin of the patients, the Quantum Gis geoprocessing program (QGis) was used. QGis is a free tool licensed by General Public License (GNU), based on a Geographic Information System that allows for the creation of maps. Therefore, 22 (50.00%) participants came from Embu das Artes and 22 (50.00%) from Taboão da Serra (Table 3).

**Table 3:** Distribution of patients according to origin.

Origin	Patients	Percentage (%)
Embu das Artes, SP	22	50.00
Taboão da Serra, SP	22	50.00
Total	44	100.00

Data collection was based on the analysis of medical records, adding the information to a form to compile the information pertinent to this study and organizing it in Microsoft Excel spreadsheets. Subsequently, the information was exported to the Statistic Package for Social Sciences (SPSS) statistical software program version 24.0 for descriptive and inferential statistical analysis. In this study, the data were organized in 1 × c contingency tables, based on absolute and relative frequencies. Pearson’s nonparametric chi-square test was used to evaluate the trend between nominal variables. A 5% significance level was used in all tests (p < 0.05).

## RESULTS

Table 4 shows the distribution of types of fracture found in our study, considering the number of patients and the percentage. Table 5 shows the mechanism of injury in our sample. Table 6 shows the distribution of patients with polytrauma and the specific types of treatment patients with polytrauma received.

**Table 4:** Distribution of fractures of patients with polytrauma in our study.

Type of fracture	Number	Percentage (%)
Diaphyseal fracture of the left femur	17	38.63
Diaphyseal fracture of the right femur	14	31.81
Subtrochanteric fracture of the right femur	3	6.82
Femoral neck fracture	2	4.55
Bilateral diaphyseal fracture of the femur	2	4.54
Open diaphyseal fracture of the right femur	2	4.55
Subtrochanteric fracture of the right femur	1	2.27
Fracture of the neck of the right femur + diaphyseal fracture of the right femur	1	2.27
Left femur diaphyseal fracture + iliac fracture	1	2.27
Total	44	100.00

**Table 5:** Distribution of injury mechanisms of patients with polytrauma in our study.

Fracture mechanism	Number	Percentage (%)
Fall from height	25	56.81
Vehicle collision	6	13.64
Run over	6	13.64
Motorcycle fall	2	4.55
Firearm projectile + run over	2	4.55
Aggression	1	2.27
Firearm projectile	1	2.27
Bicycle fall	1	2.27
Total	44	100.00

**Table 6:** Distribution of specific types of fracture treatment of patients with polytrauma in our study.

Treatment of fractures	Number	Percentage (%)
Linear external fastener	20	45.45
Locked intramedullary rod	9	20.45
Osteosynthesis with plate	7	15.91
*In situ* pinning with 2 cannulated screws of 7 mm	3	6.82
Flexible rod	3	6.82
Circular external fixator	1	2.27
Skeletal traction	1	2.27
Total	44	100.00

Table 7 shows patients’ hospital length of stay, considering the intervals: up to 3 days, from 4 to 10 days, and from 11 to 17 days, with a minimum period of 3 days and a maximum of 17 days. Of these, 20 (45.45%) participants remained hospitalized from 4 to 10 days, 19 (43.18%) for up to 3 days, and 5 (11.36%) from 11 to 17 days. Of all patients, 40 (90.91%) did not need to remain hospitalized in the Intensive Care Unit (ICU), and 4 (9.08%) did (Table 8).

**Table 7:** Distribution of the 44 patients according to length of hospital stay

LENGTH OF HOSPITAL STAY	Number	Percentage (%)
Up to 3 days	19	43.18
From 4 to 10 days	20	45.45
From 11 to 17 days	5	11.36

**Table 8:** Distribution of the 44 patients according to admission to the ICU.

ICU ADMISSION	Number	Percentage (%)
NO	40	90.91
YES (7 DAYS)	1	2.27
YES (6 DAYS)	1	2.27
YES (4 DAYS)	1	2.27
YES (2 DAYS)	1	2.27

ICU: intensive care unit.

Table 9 shows that 36 patients (81.81%) had no other lesions, while eight (15.89%) did not (extradural hematoma + pneumomediastinum, pneumothorax + pneumocephalus, hemosinus + severe head trauma, hemopneumothorax, severe head trauma, pulmonary contusion + hemorrhagic shock, extradural hematoma, hemothorax).

**Table 9:** Distribution of the 44 patients according to the associated lesions.

ASSOCIATED INJURIES	Number	Percentage (%)
No	37	84.11
Extradural hematoma + pneumomediastinum	1	2.27
Pneumothorax + pneumocranium	1	2.27
Hemosinus + severe head trauma	1	2.27
Hemopneumothorax	1	2.27
Severe head trauma	1	2.27
Pulmonary contusion + hemorrhagic shock	1	2.27
Extradural hematoma	1	2.27
Hemothorax	1	2.27

Table 10 shows the 44 patients according to the main outcomes. Of these, 34 (77.27%) did not need to be reoperated while 10 (22.73%) were surgically treated again. Regarding patients with femoral diaphyseal fracture, 12 (27.29%) used an external fixator to stabilize their lesions and all implants were removed during treatment; 5 (11.36%) had the conversion of the external fixator to intramedullary rod; 4 (9.09%) needed to remove the intramedullary rods; one (2.27%) required conversion to plate (bilateral); one (2.27%) had conversion to rigid rod; one (2.27%) had loss of reduction and revision with rod; one (2.27%) had to remove the fixator and an osteotomy was performed; one (2.27%) had a clinical hospitalization; one (2.27%) had osteonecrosis of the femoral head and the screws had to be removed; one (2.27%) had to remove the plate. No deaths were recorded when considering the treatments performed and the associated lesions.

**Table 10:** Distribution of the 44 patients according to the main outcomes

OUTCOME	Number	Percentage (%)
No reoperation	34	77.27
Removal of the external fastener	20	45.45
Conversion to rod	5	11.36
Rod removal	4	9.09
Removal of plate	1	2.27
Conversion to rigid rod	1	2.27
Conversion to plate (bilateral)	1	2.27
Removal of the external fastener	1	2.27
Loss of reduction and revision with rod	1	2.27
Osteonecrosis of the femoral head	1	2.27
Clinical admission	1	2.27
Mortality	0	0.00

## DISCUSSION

In our study, most trauma cases involved children aged from 12 to 14 years, most commonly presenting falls from heights and automobile accidents as injury mechanisms, similar to other studies in the literature. ^(^ ^10^ ^)-(^ ^12^

This etiological factor is significantly high in developing countries, as children are more exposed to this risk. We emphasize that a large circulation of vehicles, in association with a reduced socioeconomic condition, absence of education in traffic safety, and the lack of available safety equipment aggravate these risks to the population. ^(^ ^11^ ^),(^ ^12^

The predominance of accidents involving males can be culturally justified by the activities performed by them that involve high speed, force, body impact. Thus, males are significantly more exposed to this type of accidents.

A study that aimed to evaluate the profile of pediatric hospitalizations due to external causes reported that 73% of the victims were male. Moreover, the study showed that the largest number of cases (32%) occurred in individuals aged from 10 to 12 years, followed by those aged from 4 to 6 and 7 to 9 years. ^(^ ^13^ These findings are similar to those obtained in our study.

The primary objective of our study was to evaluate the clinical and surgical outcomes of patients diagnosed with polytrauma associated with severe fractures of the locomotor system (femur and pelvis).

We obtained essential information regarding our sample’s characteristic. According to our findings, all patients were discharged from hospital. Furthermore, the most common type of fracture was the closed diaphyseal of the femur in 31 (70.44%) patients. The polytrauma correlation associated with fracture of the pelvis, proximal femur, and femoral diaphyseal showed that 37 (84.11%) patients had no associated lesions. Regarding the most common fracture mechanism, we found 25 (56.81%) falls from height, 6 (13.64%) vehicle collisions, and 6 (13.64%) run overs. Considering the length of hospital stay, 19 (43.18%) patients stayed for the minimum period of 3 days, 20 (45.45%) patients from 4 to 10 days. In total, 40 (90.91%) patients did not need to be admitted to the ICU.

Femoral diaphyseal fractures represent 1.5% of all injuries involving the immature locomotor system^14^ and are related to vehicle collisions and sports accidents. These fractures require prolonged treatment in the hospital and most often surgical intervention, which socioeconomically affects the lives of children and their families. ^(^ ^15^

The early surgical stabilization of these fractures, in the first days after the injury, reduces the length of hospitalization and the length of ICU stay. Moreover, the surgery decreases the time required for assisted ventilation and significantly decreases the rate of complications. In this study, most patients received early surgical intervention, with a clear short time between hospitalization, treatment, and discharge, as the literature recommends.

Among many treatments available for femoral diaphyseal fractures, the evolution of implantable devices such as the use of the flexible intramedullary rod and the use of external fixators and plates and screws is remarkable. ^(^ ^16^ ^),(^ ^17^ In our casuistry, these implants were used.

Pelvic injuries in children, although unusual, result from high-energy trauma and are associated with severe multisystem injuries and high mortality rates. Few fractures are treated surgically, as most of them do not compromise the biomechanical stability of the pelvis. By improving knowledge about the repercussions of trauma to the pelvis of children and adolescents, many advances in minimally invasive surgical techniques have improved comfort, reducing complications. In our casuistry, we found only one (2.27%) patient with pelvic injury, who had an isolated and stable fracture of the iliac that did not provide anatomical, biomechanical, or hemodynamic alteration.

Polytrauma is the leading cause of mortality in adolescents and children. Smaller stature and lower body weight are determinants for the severity of the injury in children. The pattern of injury in polytrauma among children is directly dependent on age. Until school-age traumatic brain injury (TBI) is the most common injury. The extremities, chest, and abdomen are more frequently affected in older children in which, particularly thoracic and cranial trauma, are considered essential predictive factors to determine the outcomes of these patients with polytrauma.

Due to these differences in anatomy and physiology, pediatric patients with polytrauma require early diagnoses adjusted for age-specific variations. The first hour after the injury is considered the most critical in the care of polytrauma and directly determines the mortality rates. The first medical action is the evaluation whether the trauma is life-threatening or not. Then, the general picture of the patient must be stabilized.

Early estimation of the severity of the injury and the location of the affected organs is supported by the use of specific diagnostic imaging tools, including radiography, computed tomography, and whole-body ultrasound.

Markers for organ injury for severe trauma often point to involvement of the locomotor apparatus as well as internal organs. The early post-trauma inflammatory response induces secondary organ damage. Furthermore, organ damage is characterized by systemic elevation of specific biomarkers, such as in the heart, kidneys, and liver, where several well-established laboratory biomarkers determine their impairment. However, brain, lung, and splenic lesions are diagnosed by advanced imaging techniques.

Head trauma is the leading cause of mortality among adolescents and children. For pediatric management, neuroimaging is used to improve the child’s clinical care. To properly estimate brain injury, CT and MRI scans are used to immediately detect hemorrhage, acute hydrocephalus, fractures, and other intraparenchymal intracranial lesions. Other than neuroimaging techniques, plasma biomarkers can be a reliable tool for the clinical evaluation of pediatric TBI.

Many laboratory markers have recently been described to support the early diagnosis and prognosis of severely injured children. However, the combination of imaging techniques and a reliable prognostic laboratory biomarker can improve the rapid and adequate assessment of pediatric injuries after trauma and improve the outcome of children with polytraumatism.

Despite the higher frequency of TBI in children, compared to orthopedic injuries in adults, the ability to recover from central nervous system injuries is much greater. The movement in the fracture focus of a long bone increases intracranial pressure, which makes it necessary to early stabilize the fracture focus, determining the use of immediate internal or external osteosynthesis, facilitating care and patient transportation. Bone instability keeps the patient immobile and interferes with the care of comorbidities and brain, thoracic, and abdominal traumas. This instability also limits the intensive care of the nursing and physical therapy service.

The direct costs of pediatric trauma exceed eight billion dollars. This estimative considers only the direct costs with the approach to pediatric patients with polytrauma, since the indirect costs that affect families and society cannot be estimated. In Brazil, according to DATASUS, the total expenditure in 2005 for the clinical surgical specialty was approximately three billion reais, which shows the economic contrast for health in our country.

Regarding patients who required hospitalization, the average amount paid per hospital stay, according to data from 2015, was $ 562.00 for the Southeast region. Considering the total number of patients, although 19 of them had length of stay equal to or less than 3 days, 20 (45.45%) from 4 to 10 days, and 5 (11.36%) from 11 to 17 days. Considering the estimated daily value of a ward hospitalization as $ 206.87 reais, the average cost of the hospitalization period ranged from 827.48 to $ 2068.70. Regarding the costs of ICU hospitalization, the final cost is even higher. Although only four patients were admitted to an ICU, ranging from 2 to 7 days, the daily value of ICU hospitalization was estimated at $ 656.71. Thus, the values required by patients during their ICU stays ranged from 1313.42 to $ 4596.97. Thus, these values make patients with polytrauma hospitalization much more expensive.

Determining indicators in polytrauma allows for the application of prevention measures by continuing education. The orthopedist participates both in the diagnosis and in the treatment, as well as disseminating this information. The implementation of an efficient system of awareness and preventive education of the population, related to safety, based on the results of the epidemiological studies carried out, should be considered essential. Notably, based on the main trauma mechanisms found in our study, we emphasize the relevance of traffic education, the mandatory use of seat belts, the transport of young children with special vehicular devices, the transport of children in the rear seat, the mandatory use of protective helmets when traveling with a motorcycle, the use of protective items such as gloves and knee pads when riding a bicycle or skateboard, the supervision of adults, among other measures. ^(^ ^18^ There is a need to maximize efforts in trauma prevention and improve primary care and health care services by providing high-quality trauma care.

Our study has many limitations since it was conducted in a single medical center and is retrospective. However, over 10 years, all records of patients with polytrauma associated with severe fractures were obtained, which could have determined an unfavorable outcome increasing the mortality rate.

## CONCLUSION

In total, 34 (77.27%) patients did not need to be reoperated; 10 (22.73%) underwent a new surgical approach. Regarding the patients with femoral diaphyseal fracture, 20 (45.45%) used an external fixator to stabilize their lesions and all implants were removed during the treatment; five (11.36%) had the conversion of the external fixator to intramedullary rod; four (9.09%) needed to remove the intramedullary rods; one (2.27%) required conversion to plate (bilateral); one (2.27%) had conversion to rigid rod; one (2.27%) had loss of reduction and revision with rod; one (2.27%) had to remove the fixator with and perform an osteotomy; one (2.27%) had a clinical hospitalization; 1 (2.27%) had osteonecrosis of the femoral head and the screws had to be removed; one (2.27%) had to remove the plate.

No deaths were recorded when considering the treatments performed and the associated lesions.
